# Rationale, development and feasibility of a national prehospital transfusion registry

**DOI:** 10.1016/j.resplu.2025.101211

**Published:** 2026-01-04

**Authors:** Nura Khattab, Noah Zweig, Mahvareh Ahghari, Luis Da Luz, Melissa McGowan, Michael Peddle, Harley Meirovich, Aditi Khandelwal, Yulia Lin, Brodie Nolan

**Affiliations:** a Faculty of Medicine, University of Toronto, Toronto, Ontario, Canada; b Division of Transfusion Medicine and Tissue Bank, Precision Diagnostics and Therapeutics Program, Toronto, Ontario, Canada; c Department of Laboratory Medicine and Pathobiology, University of Toronto, Toronto, Ontario, Canada; d Ornge, Mississauga, Ontario, Canada; e Division of General Surgery, Tory Trauma Program, Sunnybrook Health Sciences Centre, Toronto, Ontario, Canada; f Department of Emergency Medicine, St. Michael’s Hospital, Toronto, Ontario, Canada; g Division of Emergency Medicine, Department of Medicine, Western University, London, Ontario, Canada; h Division of Hematology, Department of Medicine, St. Michael’s Hospital, Toronto, Ontario, Canada; i Department of Medicine, University of Toronto, Toronto, Ontario, Canada; j Canadian Blood Services, Ottawa, Ontario, Canada; k Division of Emergency Medicine, Department of Medicine, University of Toronto, Toronto, Ontario, Canada

**Keywords:** Prehospital, Trauma, Transfusion, Registry

## Abstract

**Background:**

Out-of-hospital blood transfusion (OHBT) is an emerging practice for the management of hemorrhagic shock following trauma. The Canadian Prehospital and Transport Transfusion (CAN-PATT) network aims to standardize OHBT practices and assess the feasibility of linking out-of-hospital care with in-hospital outcomes through a national registry.

**Methods:**

This was a retrospective cohort study of patients who received OHBT through an air ambulance program between September 2021 and July 2024 and were transported to one of two regional trauma centers. Prehospital data from the air ambulance database were linked using indirect identifiers to hospital data from the trauma registries and manually reviewed charts. The primary outcome was the percentage of prehospital and in-hospital records that could be successfully linked. Continuous variables were summarized as means/standard deviations or medians/interquartile ranges, and categorical variables as counts and frequencies.

**Results:**

There were 96 patients who received an OHBT during the study period; 90 were transported to a participating regional trauma center and 6 died prior to transport. Of the 90 patients, 82 (91 %) were successfully linked (Site 1: 36/39; Site 2: 46/51) between the air ambulance database and hospital trauma registries using indirect identifiers (age, sex, date and time of transport).

**Conclusion:**

This study demonstrates the feasibility of linking prehospital and in-hospital records for OHBT recipients, achieving a 91.1 % linkage rate. Future work should aim to incorporate trip numbers and missing variables into hospital registries to support the establishment of a national OHBT registry to enhance prehospital trauma care.

## Introduction

Trauma-related hemorrhagic shock is a leading cause of preventable death worldwide, contributing to approximately 1.9 million fatalities annually.^1^ Effective management of hemorrhagic shock necessitates rapid control of bleeding and resuscitation to restore intravascular volume and oxygen-carrying capacity, thereby mitigating shock severity.^2^, ^3^ Out-of-hospital blood transfusion (OHBT) has emerged as a promising intervention to address trauma-related hemorrhagic shock in the prehospital setting. ^4^ Although OHBT is becoming widely adopted in military and civilian trauma care, there is significant OHBT practice variability.^3^ A survey conducted by the European Society of Anaesthesiology revealed substantial inconsistencies in the type of blood products used, transfusion indications, and adjunctive therapies.^5^

Recognizing the need for standardization, the Canadian Prehospital and Transport Transfusion (CAN-PATT) Network was created to collaborate, synchronize, and evaluate the effectiveness of OHBT protocols across Canada.^3^ The network is comprised of six critical care transport organizations across Canada with 30 bases, including rotor-wing, fixed-wing, and land transport resources (19 rotor-wing, 20 fixed-wing, and 6 land).^3^ Among these, 11 have active blood-on-board programs. Blood-on-board programs enable medical teams to depart without delay, reducing the time to blood product administration for patients in critical need. Each organization is responsible for maintaining its own OHBT infrastructure and training programs, as well as collecting standardized data for quality assurance and research initiatives. Since the establishment of CAN-PATT, there has been a strong alignment between expert recommendations and current practices, with an observed 89 % adherence to OHBT guidelines among Canadian critical care transport organizations^6^

A major objective of CAN-PATT is to create a national OHBT registry to systematically link prehospital transfusion data with in-hospital outcomes.^3^ Traditionally, trauma registries have been used to evaluate the effect of organized trauma systems, yet they often lack comprehensive prehospital data.^7^ They may exclude critically injured patients who die during transport or shortly after arrival.^7^ The establishment of an OHBT registry would address these gaps by uniting prehospital and hospital data to allow robust outcome analyses. Such a registry could help address high-priority research questions generated by the National Trauma Research Action Plan panel on prehospital and mass casualty trauma care.^8^ Many of these high-priority questions focus on identifying OHBT practices that reduce mortality, determine ideal candidates for OHBT, and evaluate its efficacy in rural and remote populations.^8^

For these reasons, the primary objective of this study is to describe the rationale, development, and feasibility of a national out-of-hospital blood transfusion registry. This will be accomplished by exploring the feasibility of linking prehospital and in-hospital records for patients who were recipients of the blood-on-board program at Ornge, a provincial air ambulance service. Understanding the challenges and success rates of data linkage will provide valuable insights into the practical considerations of establishing a nationwide registry, ultimately contributing to improved trauma care and patient outcomes. By capturing the entire prehospital-to-in-hospital trajectory, a coordinated OHBT registry can enable earlier recognition of effective transfusion strategies, support protocol optimization, and identify survival-associated factors to facilitate real-time, evidence-based improvements in transfusion practices.

While international linkage efforts exist, Canadian OHBT systems span vast geography, rely heavily on aeromedical transport, and manage prolonged prehospital intervals, limiting the applicability of international findings. This study therefore aims to provide context-specific Canadian insights.

## Methods

### Design

We conducted a retrospective cohort study to examine patients who received at least one unit of OHBT through the air ambulance blood-on-board program operated by Ornge, from August 2021 to July 2024 and transported to one of two Level 1 trauma hospitals, Sunnybrook Health Sciences Center (SHSC) or St. Michael’s Hospital (SMH)/Unity Health Toronto.

### Setting

Ornge, the provincial air ambulance service operating in Ontario, Canada, launched its OHBT program on August 31, 2021 at its Toronto rotor-wing base (799). Ornge receives approximately 20,000 calls annually, with most patients receiving OHBT being trauma patients.^3^ While the air ambulance service transports patients to nine adult trauma centers and four pediatric trauma centers across the province, a large proportion of severely injured patients transported by 799 are transferred to one of two adult lead trauma hospitals: SHSC and SMH. SHSC is a 1200-bed tertiary care hospital with an annual emergency department (ED) volume of 61,000 visits, of which over 1600 are trauma-related cases with an Injury Severity Score (ISS) of 15 or greater.^3^ SMH, a 500-bed tertiary care hospital, handles 75,000 ED visits annually, with 1100 trauma cases meeting an ISS of 15 or greater.^3^ Focusing on these two high-volume receiving centers allows the evaluation of linkage feasibility within the primary clinical catchment for sites adopting early OHBT practices while minimizing variability in documentation processes during this foundational pilot phase.

### Population

All patients who received at least one unit of OHBT from Ornge during the study period were identified using Ornge's health administrative database. Patients who received at least one unit of blood in the prehospital environment and were transported to either SHSC or SMH were included in the study. This sample, representing the first two-and-a-half years of the program, allowed for an assessment of the feasibility of linking prehospital and hospital records.

### Development of data elements

Based on expert consensus from the CAN-PATT Network, a comprehensive set of variables were agreed upon for inclusion in a national OHBT registry. These variables encompass data collected by both critical care transport organizations (e.g., Ornge) and receiving hospitals (e.g., SHSC/SMH). To organize the data within the registry, the variables were categorized into two distinct phases of care: prehospital and in-hospital. Prehospital data included patient demographics (age, sex), bleeding etiology (traumatic vs. non-traumatic), transport details (mode, type), call events (arrival and transport times, pre-transfusion vital signs), and blood products transfused. In-hospital data covered call events (time of transfer of care and arrival), the activation of the massive hemorrhage protocol, blood products transfused, initial vital signs, clinical interventions (angiography, endoscopy), and post-discharge outcomes (disposition, discharge time). A detailed list of included prehospital and hospital variables, and description of each variable is outlined in Appendix 1.

### Case identification, data capture, and integration of disparate data sources

Prehospital data was collected from Ornge’s health administrative database, which compiles information from the paramedic electronic patient care record (ePCR) and the dispatch software (FlightVector). These records include patient demographics, vital signs, clinical data, and interventions performed by paramedics. In-hospital data was retrieved from patient charts and the hospital-based trauma registries at SHSC and SMH, which prospectively collect patient demographics, injury, and hospital-resource data during hospitalization.

To assess the feasibility of linking prehospital and in-hospital records, patient data from Ornge was matched to the corresponding trauma registry entries at SHSC and SMH. Because of initial restrictions on the use of personal health information in this research study, patients were linked between the Ornge transport database and the trauma registries using indirect identifiers, including sex, age, arrival date and time, and confirmation of Ornge transport. A 45-min time window was applied to account for differences between Ornge handover times and trauma registry ED arrival times. Minor age discrepancies were permitted to account for estimation in unidentified patients. All identifiers were used collectively to maintain rigorous and accurate patient matching, ensuring correct linkage between the datasets. Research personnel conducted the linkage process using predefined matching criteria to optimize accuracy and limit data discrepancies. Following the discovery that complete linkage could not be achieved with indirect identifiers alone, the Ornge Ambulance Call Report (ACR) number was subsequently incorporated post hoc (and with research ethics board (REB) approval) at one of the hospital sites where this information was collected to determine if remaining unmatched cases could be linked.

**Fig. 1:**
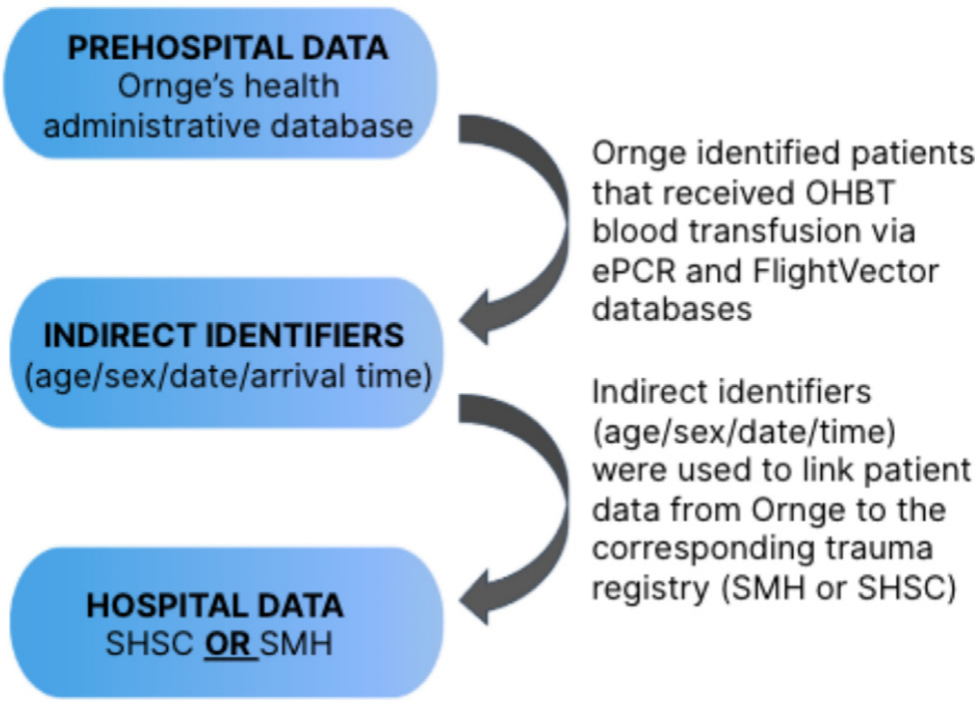
**Study data flow diagram**.

The data collection process followed a structured workflow, quantifying the percent variables available in the Ornge ePCR and trauma registries. Remaining variables were collected using manual extraction from the hospital charts to ensure comprehensive data inclusion. The overall data flow is depicted in Fig. 1.

### Outcomes

The primary outcome of this pilot study was the proportion of patients who received OHBT through Ornge and were transported to SHSC or SMH, with successfully linked prehospital and in-hospital records. Secondary outcomes included the percentage of registry variables collected by existing registry vs. manual chart review. Additional secondary clinical outcomes included a detailed description of the patient population. This encompassed patient demographics, injury patterns, blood consumption, need for further blood transfusions in hospital, and in-hospital mortality.

### Data analysis

Descriptive statistics were used to evaluate the distribution of data for all variables of interest in this study. Categorical variables were summarized as counts and frequencies. In particular, the linkage rate was calculated as a proportion. Moreover, continuous variables were summarized using either means and standard deviations (for normally distributed data) or medians and interquartile ranges (for non-normally distributed data). All statistical analyses were conducted using Microsoft Excel 2016.

### Ethical considerations

Ethical approval was obtained from the REB through Clinical Trials Ontario (CTO) (Project ID: 4541). Pre-existing REBs and data-sharing agreements between Ornge, SHSC, and SMH facilitated the sharing of research data.

## Results

### Linkage rate

Between August 2021 and July 2024, a total of 96 patients received at least one unit of OHBT through the Ornge blood-on-board program. Of these, 90 patients were subsequently transported to either SMH or SHSC, while 6 patients died prior to transport which led to their omission from the study population. Of the final cohort of 90 patients, 82 (91.1 %) were successfully linked to the corresponding in-hospital records. Specifically, linkage was achieved for 46 out of 51 patients (90.2 %) at SMH and 36 out of 39 patients (92.3 %) at SHSC.

A total of 29 in-hospital variables were identified for extraction at each site. Of these, 13 variables were available from the SHSC trauma registry and 15 from the SMH trauma registry. The remaining 16 variables at SHSC and 14 at SMH were retrieved through manual chart abstraction.

### Patient Characteristics

The study population was summarized by analyzing patient demographics (age, sex, injury patterns), blood product utilization, vital signs, and in-hospital outcomes (Table 1). 76 % of patients in this study were male, and the median age was 40 (IQR 28–58). Injuries were predominantly blunt (90 %) and only 9 % of cases presented with penetrating trauma. At the scene, the median Glasgow Coma Scale (GCS) was 6 (IQR 3–14), with documented improvement upon hospital arrival (median 13; IQR 3–15). Approximately 28 % of patients were intubated prior to or during transport.

**Table 1:** Study demographics.

**Patient characteristics**	**Total cohort** ***n* = 90**
Age, years, *median (IQR)*	40 (28–58)
**Sex, *n (%)***	
Male	68 (76%)
Female	22 (24%)
**Mechanism of injury, *n (%)***	
Blunt	81 (90%)
Penetrating	8 (9%)
Blunt + Penetrating	1 (1%)
**Transport time characteristics in minutes, *median (IQR)***	
Time from call accept to arrival at patient	52 (40–69)
Time from arrival at patient to arrival at trauma centre	77 (60-97)
**Vital Signs, *median (IQR)***	
Prehospital systolic blood pressure, mmHg	85.5 (71–102)
Prehospital heart rate, beats per minute	97 (76–124)
Prehospital respiratory rate, breaths per minute	20 (17–25)
Prehospital oxygen saturations	96 (90–99)
Prehospital Glasgow Coma Scale	6 (3–14)
Prehospital temperature (°C)	35.6 (34.7–36.1)
In-hospital systolic blood pressure, mmHg	116 (99–133)*
In-hospital heart rate, beats per minute	95.5 (78–100)*
In-hospital respiratory rate, breaths per minute	21 (18–25.5)*
In-hospital oxygen saturations	99 (96–100)*
In-hospital Glasgow Coma Scale	13 (3–15)*
In-hospital temperature (°C)	35.4 (34.6–36.3)*
**Blood and blood products administered *median (IQR)***	
Prehospital red blood cells, units	2 (1–2)
Prehospital plasma, units	0 (0–0)
Prehospital platelets, doses	0 (0–0)
Prehospital fibrinogen, grams	0 (0–0)
Prehospital prothrombin complex concentrates, units	0 (0–0)
In-hospital red blood cells, units	2 (0–5)**
In-hospital plasma, units	0 (0–4)**
In-hospital platelets, doses	0 (0–0)**
In-hospital fibrinogen, grams	4 (0–11)**
In-hospital prothrombin complex concentrates, units	0 (0–0)*
**In-hospital outcomes, *n (%)***	
In-hospital massive hemorrhage protocol activation	41 (50%)*
Surgical hemorrhage control	29 (35%)*
Interventional radiology	20 (24%)*
In-hospital mortality	18 (22%)*

* *N* = 82 (82/90 patients were linked).

** *N* = 78 (4 patients had unreported units for RBC, FFP, PLT, FGY).

### Prehospital clinical parameters

The median transport time from call to hospital arrival was 138 min (IQR 108–162 min) (Table 1).

### Transfusion patterns and blood product utilization

Detailed characteristics of blood product utilization are outlined in Table 2. Among the 90 patients, Ornge administered a total of 224 blood products during transport, comprising 205 units of RBCs and 19 units of plasma. Of the 82 patients successfully linked to in-hospital data, the massive hemorrhage protocol (MHP) was activated within one hour of arrival in 49.4 % of cases (41 out of 82). During the first 24 h of hospitalization, patients received 363 units of RBCs, 211 units of plasma, 35 units of platelets, 597 g of fibrinogen concentrate, and 13 doses (500 international units each) of prothrombin complex concentrate. Seventeen patients (20.7 %) did not receive any blood products during the first 24 h of hospitalization. Among those who did receive blood products, the median number of units transfused per patient was 13 (IQR 5–23). Four patients did not have their in-hospital blood utilization reported, and ten patients accounted for 43 % of the total blood products transfused in this study.

**Table 2:** Blood product utilization.

**Blood product**	**Units transfused by Ornge (prehospital)**	**Units transfused in first 24 hours (In-hospital)**
RBCs (units)	205	363
Plasma (units)	19	211
Platelets (doses)	0	35
Fibrinogen (g)	0	597
PCC (doses*)	0	13
Whole Blood (units)	0	0

* 1 dose = 500 international units.

### Hospital outcomes

The overall in-hospital mortality rate was 22.0 %. A significant proportion of patients were discharged to rehabilitation (23.2 %) or transferred to another hospital (25.6 %), highlighting the severity of injuries sustained. Twenty-eight percent were discharged home, and the remaining 1.2 % to long-term care facilities.

### Post-hoc data linkage

Given the incomplete nature of the initial data linkage, additional methods were explored post-hoc. With REB approval, permission was obtained to utilize the Ornge ACR number (trip number), which was documented at the SHSC site but not at SMH. Utilization of this identifier allowed for the successful linkage of the remaining cases. Furthermore, the ACR number was used to validate the accuracy of cases that had previously been linked using indirect identifiers, with no false positives observed.

## Discussion

This pilot study sought to explore the feasibility of establishing a national OHBT registry in Canada, aiming to inform and support the future integration of OHBT into standardized trauma systems. The study yielded four major findings. First, the technical and methodological feasibility of linking prehospital and in-hospital data was possible. Second, challenges exist in linking trauma registries due to heterogenous data collection and incomplete or inconsistent data elements. Third, a small subset of patients accounted for a disproportionately large share of transfusions, highlighting substantial variation in transfusion requirements across patients. Fourth, patients receiving OHBT within Canada have prolonged out of hospital times compared to those seen in the US and Europe.

OHBT in trauma represents an evolving frontier in prehospital care, with growing recognition of its potential to bridge the critical time gap between injury and definitive hemorrhage control. By allowing earlier resuscitation, OHBT holds significant promise in decreasing preventable mortality among trauma patients.^3^, ^4^, ^6^ To fully understand and evaluate this impact, robust data linkage is essential.

### Data linkage feasibility

Accurate data linkage enables researchers and clinicians to follow patients along the continuum of care, from initial prehospital management through to definitive in-hospital treatment. In this study, initial linkage using indirect identifiers alone resulted in a match rate of 91.1 %, highlighting the limitations of relying solely on non-specific variables. Among the eight unlinked cases, linkage failures were largely due to discrepancies in patient age and mismatches in recorded arrival times. Age is often estimated prehospital, creating inconsistencies with hospital records. Successful linkage also requires air ambulance and hospital arrival times to align within ±15 min. Variability and estimation in the recording of arrival times likely contributed to these linkage failures. Standardized, coordinated documentation between air ambulance crews and hospital staff may reduce these discrepancies and improve linkage.

Preliminary analysis of incorporating the patient specific Ornge ACR number shows that complete linkage of all cases could be achieved. This was demonstrated at the SHSC site. At present, the ACR number cannot be readily retrieved from the electronic patient record system at SMH and is not recorded within the trauma registry. Taken together, these findings underscore the importance of routinely capturing the ACR number in hospital trauma registries and records to facilitate complete and accurate linkage of prehospital and in-hospital data.

Our findings are consistent with prior work by Newgard et al.^9^ and Blanchard et al.,^10^ who validated probabilistic linkage methods for combining EMS and trauma registry data across multiple systems in the United States. These studies demonstrated that even in the absence of unique patient identifiers, linkage algorithms can achieve high match rates and support large-scale trauma research. Newgard et al. reported match rates as high as 98 %, while Blanchard et al. reported rates up to 95 %.^9^, ^10^ Complementing this work, the PROMMTT pilot study demonstrated feasibility for similar integration in Canada.^11^ This linkage has the potential to overcome data silos, enabling integrated datasets that enhance trauma research and improve evaluation of time-sensitive prehospital interventions.^10^, ^11^, ^12^, ^13^, ^14^

### Limitations of registry linkage

Challenges in linking trauma registries arise from heterogeneous data collection procedures and incomplete or inconsistent data elements, which are well documented in the literature. The development of successful trauma registries in the United Kingdom and United States were accompanied by challenges that parallel those observed in this study.^15^, ^16^, ^17^ Of the 29 requested variables, less than half were routinely collected as part of standard clinical documentation, necessitating extensive manual chart review to collect the remaining data. Another challenge identified in our study was that the two lead trauma centers differed in the types of variables collected and the formats in which data were recorded. For example, date fields were formatted inconsistently across sites (MM/DD/YYYY vs. DD/MM/YYYY), requiring manual reconciliation prior to dataset integration. These variations underscore the ongoing need for standardization in trauma data collection across centers to facilitate streamlined analysis and system-wide quality improvement.

Linking patient records also posed challenges. In compliance with privacy regulations, personal health identifiers such as provincial health insurance numbers and patient names were not used. Instead, linkage initially relied on indirect identifiers including age, sex, and arrival time. While this method enabled high-confidence matching in most cases, discrepancies in age estimation and/or time-stamping across datasets introduced uncertainties that required confirmation using contextual factors and chart review. Complete matching and accuracy were achieved at one site once post-hoc Ornge ACR numbers were incorporated.

The limited sample size represents an additional limitation of this pilot study. The 90-patient cohort was derived from two lead trauma hospitals within a single province. While these centers are among the largest and most advanced in Canada, they may not reflect practices in smaller or rural centers, thereby limiting generalizability. Although the 91.1 % linkage rate is encouraging, it was achieved through retrospective manual review at two sites with similar documentation systems and shared provincial regulations. National implementation will require addressing interprovincial variability in privacy legislation, heterogeneity in EMS–hospital record structures, and the substantial personnel demands associated with manual linkage and data abstraction. Establishing a sustainable national OHBT registry will therefore necessitate coordinated investment, harmonized data standards, and automated linkage processes.

### Transfusion characteristics

Analysis of patient-level transfusion patterns revealed that a small subset of patients accounted for a disproportionately high volume of in-hospital blood product use. Specifically, 11 % of the study cohort received 43 % of all transfusions administered. Notably, as a collective, these patients did not experience longer prehospital transport times or lower GCS scores, indicating that transfusion requirements were not solely explained by delayed transport or overt neurologic compromise. Furthermore, all six patients who received exceptionally high fibrinogen doses were treated at the same institution, suggesting potential variability in institutional transfusion practices. Fibrinogen doses among these cases ranged from 32 to 68 g, compared with a maximum of 12 g per patient at the other site. These findings highlight both the heterogeneity of transfusion practices across centers and the importance of considering patient- and institution-level factors when interpreting transfusion requirements in prehospital trauma care.

### Transport characteristics

In this pilot study, the median transport time from call to hospital arrival was 138 min, substantially exceeding the out-of-hospital intervals reported in major prehospital transfusion trials. The COMBAT trial in the United States reported a median scene-to-hospital time of 18 min, while the PAMPER and RePHILL trials in the United Kingdom reported transport times of 41 min and 83 min, respectively.^4^, ^18^ These prolonged transport times likely reflect the unique geographic challenges, reliance on aeromedical transport, and longer transfer distances characteristic of the Canadian trauma system. The type of calls can also influence transport times. Modified calls, involving interfacility transfers or delayed dispatch, often result in longer transport durations compared to scene calls, which involve direct response to the injury location.

These differences in transport intervals have implications for both the potential benefit and the operational demands of OHBT in Canada. Extended out-of-hospital intervals may heighten the potential benefit of early hemostatic resuscitation, as patients spend substantially longer without access to definitive hemorrhage control compared with urban systems in the United States and Europe. However, longer transport durations also introduce logistical challenges, including increased onboard blood product requirements, risk of wastage, and more complex storage and handling needs during aeromedical missions. Additionally, they impose greater personnel demands, as crews must monitor transfusion response and manage complications over extended periods.

Accordingly, the efficacy of prehospital transfusion must be evaluated within the Canadian context, as findings from international trials may not directly relate. The ongoing Study of Whole Blood in Frontline Trauma (SWiFT) is a randomized controlled trial designed to evaluate the feasibility of prehospital transfusion research within the Canadian environment, which can help to address this gap.^19^

## Conclusion

The prehospital environment offers a pivotal window to deliver life-saving hemostatic therapy before definitive control can be achieved in-hospital. Our study confirms that linking prehospital transfusion data with in-hospital outcomes is both technically feasible and highly reliable, achieving a 91.1 % match rate across two Level 1 trauma centers with indirect identifiers alone, and up to 100 % when the Ornge ACR number was included. This proof-of-concept lays the foundation for a national OHBT registry that can systematically capture patient trajectories, shed light on real-world transfusion patterns, and drive continuous quality improvement across Canada’s trauma system.

To advance this work, routine capture of the Ornge ACR number within hospital trauma registries represents a critical and immediately actionable improvement to support record linkage. Defining a minimum set of standardized data elements is also required for national scalability, including core demographic variables, time-stamped prehospital interventions, transport intervals, and in-hospital outcomes. Establishing these requirements, alongside automated linkage processes will allow future efforts to expand this feasibility study into a sustainable, Canada-wide OHBT registry.

## Financial support

Canadian Blood Services – Blood Efficiency Accelerator Program.

## CRediT authorship contribution statement

**Nura Khattab:** Writing – review & editing, Writing – original draft, Investigation, Formal analysis, Data curation. **Noah Zweig:** Writing – review & editing, Writing – original draft, Methodology, Investigation, Formal analysis. **Mahvareh Ahghari:** Writing – review & editing, Resources, Project administration, Methodology, Investigation, Data curation. **Luis Da Luz:** Writing – review & editing, Resources, Conceptualization. **Melissa McGowan:** Writing – review & editing, Supervision, Resources, Project administration, Methodology, Investigation, Formal analysis, Data curation. **Michael Peddle:** Writing – review & editing, Resources, Methodology, Data curation. **Harley Meirovich:** Writing – review & editing, Methodology, Investigation, Data curation. **Aditi Khandelwal:** Writing – review & editing, Project administration, Data curation. **Yulia Lin:** Writing – review & editing, Supervision, Resources, Project administration, Methodology, Investigation, Conceptualization. **Brodie Nolan:** Writing – review & editing, Supervision, Resources, Project administration, Methodology, Investigation, Data curation, Conceptualization.

## Declaration of competing interest

There are no conflicts of interest to declare. YL has received research funds from Octapharma and Canadian Blood Services, honoraria from Pfizer and is a consultant with Choosing Wisely Canada. BN, MP and LDL have received honoraria from Octapharma.
