# Supplementary material for: Rationale, development and feasibility of a national prehospital transfusion registry
Source: Resusc Plus. 2026 Jan 4;28:101211. doi: 10.1016/j.resplu.2025.101211 (PMC12856417; doi:10.1016/j.resplu.2025.101211)
Supplement: Supplementary Data 1 [file mmc1.docx]

**APPENDIX 1: CAN-PATT Prehospital and Transport Blood Product Transfusion Registry Data Dictionary**

**Out-of-hospital Individual Patient Level Variables - (Collected by Critical Care Transport Organization)**

| **Variable** | **Description** |
| --- | --- |
| Age | Numerical, in years |
| Sex | Categorical:  Male, Female |
| Date of transport | Day Month Year |
| Time of transport | 24h hh:mm time; of crew arrival at patient bedside |
| Time to arrive at patient (T1) | Numerical, Time in minutes (defined as call accepted to arrival at crew arrival at patient bedside) |
| Time to transport patient (T2) | Numerical, Time in minutes (defined as time of arriving at patient bedside to arrival at receiving hospital) |
| Total time to receiving hospital (T1 + T2) | Numerical, Time in minutes  (defined as call accepted to arrival at receiving hospital) |
| Type of transport | Categorical:  Primary (Scene, hospital intercept/modified scene),  Secondary (Interfacility transport) |
| Mode of transport | Categorical:  Rotor-wing, fixed-wing, land |
| Bleeding etiology | Categorical:  Traumatic,  Non-traumatic |
| Traumatic etiology | Categorical:  Blunt,  Penetrating,  (n/a if non-traumatic) |
| Non-traumatic etiology | Categorical:  Vascular/Aneurysmal,  Gastrointestinal,  Peri- or post-operative,  Obstetrical,  Other  (n/a if traumatic) |
| Physician order obtained for transfusion prior to initiation of first unit | Categorical (yes/no) |
| Patient Consent obtained | Categorical (yes/no)  Assume emergency use consent if “no” |
| Patient identified (for reconciliation purposes) | Categorical (yes/no) |
| Group and screen obtained prior to first unit transfused | Categorical (yes/no) |
| Group and screen obtained at any time by transporting paramedics | Categorical (yes/no) |
| Pre-transfusion systolic blood pressure | Numerical  Most recent SBP taken before initiation of first unit of blood  “0” if patient in cardiac arrest |
| Pre-transfusion mean arterial blood pressure | Numerical  Most recent MAP taken before initiation of first unit of blood  “0” if patient in cardiac arrest |
| Pre-transfusion heart rate | Numerical  Most recent heart rate taken before initiation of first unit of blood  “0” if patient in cardiac arrest |
| Pre-transfusion shock index | Numerical  Calculated using above pre-transfusion SBP and HR |
| Pre-transfusion temperature | Numerical  Most recent temperature taken before initiation of first unit of blood |
| Pre-transfusion respiratory rate | Numerical  Most recent respiratory rate taken before initiation of first unit of blood  “0” if patient in cardiac arrest |
| Pre-transfusion SpO2 | Numerical  Most recent SpO2 taken before initiation of first unit of blood  “0” if patient in cardiac arrest |
| Pre-transfusion Glasgow Coma Scale | Numerical  Most recent GCS taken before initiation of first unit of blood |
| Pre-transfusion pH | Numerical  Most recent pH taken before initiation of first unit of blood (ABG or VBG)  “n/a” if unavailable |
| Pre-transfusion lactate | Numerical  Most recent lactate taken before initiation of first unit of blood (ABG or VBG)  “n/a” if unavailable |
| Pre-transfusion hemoglobin | Numerical  Most recent hemoglobin taken before initiation of first unit of blood (ABG or VBG)  “n/a” if unavailable |
| Time of initiation of transfusion | Date and time of initiation of first unit of blood  Date Month Year, 24 hh:mm |
| Total red blood cell (RBC) transfused by transporting crew (from OHBT supply) | Numerical, number of units |
| Total red blood cell (RBC) transfused by transporting crew (from hospital-based supply) | Numerical, number of units |
| Total plasma transfused by transporting crew (from OHBT supply) | Numerical, number of units |
| Total plasma transfused by transporting crew (from hospital-based supply) | Numerical, number of units |
| Total platelets transfused by transporting crew (from OHBT supply) | Numerical, number of units |
| Total platelets transfused by transporting crew (from hospital-based supply) | Numerical, number of units |
| Total fibrinogen transfused by transporting crew (from OHBT supply) | Numerical, number of g |
| Total fibrinogen transfused by transporting crew (from hospital-based supply) | Numerical, number of g |
| Total Prothrombin Complex Concentrates (PCC’s) transfused by transporting crew (from OHBT supply) | Numerical, number of IUs |
| Total Prothrombin Complex Concentrates (PCC’s) transfused by transporting crew (from hospital-based supply) | Numerical, number of IUs |
| Total whole blood transfused by transporting crew (from OHBT supply) | Numerical, number of units |
| Total whole blood transfused by transporting crew (from hospital-based supply) | Numerical, number of units |
| Post-transfusion systolic blood pressure | Numerical  Most recent SBP taken after initiation of last unit of blood  “0” if patient in cardiac arrest |
| Post-transfusion mean arterial blood pressure | Numerical  Most recent MAP taken after initiation of last unit of blood  “0” if patient in cardiac arrest |
| Post-transfusion heart rate | Numerical  Most recent heart rate taken after initiation of last unit of blood  “0” if patient in cardiac arrest |
| Post-transfusion shock index | Numerical  Calculated using above post-transfusion SBP and HR |
| Post-transfusion temperature | Numerical  Most recent temperature after initiation of last unit of blood |
| Post-transfusion respiratory rate | Numerical  Most recent respiratory rate taken after initiation of last unit of blood  “0” if patient in cardiac arrest |
| Post-transfusion SpO2 | Numerical  Most recent SpO2 taken after initiation of last unit of blood  “0” if patient in cardiac arrest |
| Post-transfusion Glasgow Coma Scale | Numerical  Most recent GCS taken after initiation of last unit of blood |
| Post-transfusion pH | Numerical  Most recent pH taken after initiation of last unit of blood (ABG or VBG)  “n/a” if unavailable |
| Post-transfusion lactate | Numerical  Most recent lactate taken after initiation of first unit of blood (ABG or VBG)  “n/a” if unavailable |
| Post-transfusion hemoglobin | Numerical  Most recent hemoglobin taken after initiation of first unit of blood (ABG or VBG)  “n/a” if unavailable |
| Transfusion reaction | Categorical (yes/no) |
| Transfusion reaction signs/symptoms | Categorical:  New fever  New hypotension  New hypoxia  New rash  Other  n/a |
| Transfusion reaction diagnosis | Categorical: Sepsis/contamination  Acute hemolytic transfusion reaction  Febrile non-hemolytic transfusion reaction  Transfusion-related acute lung injury  Transfusion associated respiratory distress  Transfusion-associated circulatory overload  Anaphylaxis  Minor allergic reaction  Bradykinin mediated hypotension  Hemolysis not related to RBC alloantibodies  Delayed hemolytic transfusion reaction  Other  Unknown  n/a |
| Transport team management of transfusion reaction | Categorical (answered as list of any below):  IV fluids  Antihistamines  Antipyretics  Steroids  Oxygen (by mask)  NIPPV  Intubation  Vasopressors  Other: |
| Fluid warmer | Categorical (yes/no)  Blood products/components given via commercial fluid warmer |
| Normothermia maintained | Categorical (yes/no)  Temperature post transfusion maintained at 35C or above |
| Tranexamic acid | Numerical, number of g,  Include dose given by sending facility (if applicable) |
| Calcium | Categorical (yes/no)  Administration of any dose of calcium gluconate or calcium chloride. Count as yes if given by sending facility staff |

**In-hospital Individual Patient Level Variables**.

| **Variable** | **Description** |
| --- | --- |
| Date of arrival at receiving hospital | Day Month Year |
| Time of arrival at receiving hospital | 24h hh:mm |
| Was a massive hemorrhage protocol (MHP) activated within 1 hour of arrival? | Categorical, yes/no |
| Total red blood cell (RBC) transfused by receiving hospital in first 24h of admission | Numerical, number of units |
| Total plasma transfused by receiving hospital in first 24h of admission | Numerical, number of units |
| Total platelets transfused by receiving hospital in first 24h of admission | Numerical, number of units |
| Total fibrinogen transfused by receiving hospital in first 24h of admission | Numerical, number of g |
| Total Prothrombin Complex Concentrates (PCC’s) transfused by receiving hospital in first 24h of admission | Numerical, number of IU |
| Total whole blood transfused by receiving hospital in first 24h of admission | Numerical, number of units |
| Receiving hospital systolic blood pressure | Numerical  First documented SBP taken on arrival to receiving hospital  “0” if patient in cardiac arrest |
| Receiving hospital mean arterial blood pressure | Numerical  First documented MAP taken on arrival to receiving hospital  “0” if patient in cardiac arrest |
| Receiving hospital heart rate | Numerical  First documented heart rate taken on arrival to receiving hospital  “0” if patient in cardiac arrest |
| Receiving hospital shock index | Numerical  Calculated using above in-hospital SBP and HR |
| Receiving hospital temperature | Numerical  First documented temperature taken on arrival to receiving hospital |
| Receiving hospital respiratory rate | Numerical  First documented respiratory rate taken on arrival to receiving hospital  “0” if patient in cardiac arrest |
| Receiving hospital SpO2 | Numerical  First documented SpO2 taken on arrival to receiving hospital  “0” if patient in cardiac arrest |
| Receiving hospital Glasgow Coma Scale | Numerical  First documented GCS taken on arrival to receiving hospital |
| Receiving hospital pH | Numerical  First available pH taken after presentation to hospital (ABG or VBG)  “n/a” if unavailable |
| Receiving hospital lactate | Numerical  First available lactate taken after presentation to hospital (ABG or VBG)  “n/a” if unavailable |
| Receiving hospital hemoglobin | Numerical  First available hemoglobin taken after presentation to hospital  “n/a” if unavailable |
| Receiving hospital Calcium | Numerical  First available calcium taken after presentation to hospital  “n/a” if unavailable |
| OR within 4h of arrival to hospital | Categorical (yes/no) |
| Interventional Angiography within 4h of arrival to hospital | Categorical (yes/no)  Ie. interventional radiology |
| Endoscopy within 4h of arrival | Categorical (yes/no) |
| Discharge disposition | Categorical (home, long term care, rehab, dead) |
| Date of discharge disposition | Day Month Year |
| Time of discharge disposition | 24h hh:mm |
